# Plasma Amino Acid Response to Whey Protein Ingestion Following 28 Days of Probiotic (*Bacillus subtilis* DE111) Supplementation in Active Men and Women

**DOI:** 10.3390/jfmk6010001

**Published:** 2020-12-23

**Authors:** Jeremy R. Townsend, William C. Vantrease, Megan D. Jones, Philip A. Sapp, Kent D. Johnson, Cheryle N. Beuning, Allison A. Haase, Claudia M. Boot

**Affiliations:** 1 Exercise and Nutrition Science Graduate Program, Lipscomb University, Nashville, TN 37204, USA; william.vantrease@lipscomb.edu (W.C.V.); mdl016@uark.edu (M.D.J.); johnsonkd@lipscomb.edu (K.D.J.); 2 Department of Nutritional Sciences, Pennsylvania State University, University Park, PA 16802, USA; pzs5199@psu.edu; 3 Central Instrument Facility, Department of Chemistry, Colorado State University, Fort Collins, CO 8052, USA; cbeuning@rams.colostate.edu (C.N.B.); Allison.Haase@colostate.edu (A.A.H.); Claudia.Boot@colostate.edu (C.M.B.)

**Keywords:** gut, microbiome, protein synthesis, leucine, absorption, BCAA

## Abstract

We sought to determine if 28 days of probiotic supplementation influenced the plasma amino acid (AA) response to acute whey protein feeding. METHODS: Twenty-two recreationally active men (*n* = 11; 24.3 ± 3.2 yrs; 89.3 ± 7.2 kg) and women (*n* = 11; 23.0 ± 2.8 yrs; 70.2 ± 15.2 kg) participated in this double-blind, placebo-controlled, randomized study. Before (PRE) and after 28 days of supplementation (POST), participants reported to the lab following a 10-hr fast and provided a resting blood draw (0 min), then subsequently consumed 25 g of whey protein. Blood samples were collected at 15-min intervals for 2 h post-consumption (15–120 min) and later analyzed for plasma leucine, branched-chain AA (BCAA), essential AA (EAA), and total AA (TAA). Participants received a probiotic (PROB) consisting of 1 x10-9 colony forming units (CFU) *Bacillus subtilis* DE111 (*n* = 11) or a maltodextrin placebo (PL) (*n* = 11) for 28 days. Plasma AA response and area under the curve (AUC) values were analyzed via repeated measures analysis of variance. RESULTS: Our analysis indicated no significant (*p* < 0.05) differential responses for plasma leucine, BCAA, EAA, or TAA between PROB and PL from PRE to POST. AUC analysis revealed no group × time interaction for plasma leucine (*p* = 0.524), BCAA (*p* = 0.345), EAA (*p* = 0.512), and TAA (*p* = 0.712). CONCLUSION: These data indicate that 28 days of *Bacillus subtilis* DE111 does not affect plasma AA appearance following acute whey protein ingestion.

## 1. Introduction

Probiotics are living organisms that when consumed provide a health benefit to the host through their effects on the intestinal tract. Administration of beneficial bacteria by the use of probiotics has been shown to promote mucosal and epithelial health of the gastrointestinal (GI) tract, providing a barrier excluding and eliminating various antigens. Evidence suggests that probiotics are an effective strategy to delay or prevent upper respiratory tract infections [1,2,3], reduce indices of GI discomfort during exercise [4], and improve carbohydrate utilization during prolonged cycling [5]. While a bulk of the literature regarding probiotics pertains to endurance athletes, data is emerging that suggests that probiotics may provide a benefit in the adaptation and recovery of athletes engaged in resistance or team sport training [6,7]. 

*Bacillus subtilis* is a rod-shaped, aerobic, Gram-positive bacterium that forms an endospore, allowing it to survive extreme temperatures and environments. *Bacillus subtilis* strains have been reported to strengthen the intestinal barrier through upregulation of tight junction proteins (TJs), reducing inflammatory cytokine release, and attenuating gut membrane barrier damage in in vitro models [8,9]. In a rodent model, *Bacillus subtilis* prevented a decrease in intestinal villi height while maintaining mucosal thickness and TJ protein expression following forced treadmill running [10]. More specifically, *Bacillus subtilis* DE111 (DE111) has been shown to survive the digestive tract [11] and promote stool regularity while reducing instances of constipation and diarrhea [12]. Previous work has shown DE111 to reduce resting tumor necrosis factor-alpha (TNF-α) concentration in athletes engaging in team sport training [13]. Furthermore, female division I athletes supplementing with DE111 in conjunction with a recovery drink for 10 weeks during offseason training reported a significant reduction in body fat percentage compared to those athletes consuming a placebo [14]. It has been suggested that the favorable results regarding probiotics and resistance exercise may be mediated by improved protein digestions and utilization via probiotic-induced modifications of GI integrity and gut microbiota [6].

Feedings high in essential amino acids (EAAs), specifically leucine, acutely stimulate muscle protein synthesis [15] and have the ability to dictate the magnitude of strength and hypertrophy adaptation following chronic resistance training [16]. However, intense physical exercise places a considerable stress on the GI tract, resulting in reduced blood flow, damage to the intestinal lining, and degradation of the mucosa [17]. Concerning resistance exercise, it has been reported that just 6 sets of the leg press and leg extension at 40–75% of the participant’s 1-repetition maximum (1 RM) resulted in significant elevations of intestinal fatty-acid binding protein (IFABP) in recreationally active males [18]. Consequently, exercise-induced GI damage has been shown to impair the absorption of both carbohydrates and protein [18,19], potentially limiting recovery and adaptation. Therefore, implementing strategies to improve the digestibility and absorption of dietary protein, through probiotic-mediated maintenance of GI function, is of interest to those engaged in physical exercise. Recently, it was shown that 14 days of supplementation with a multi-strain probiotic (5 billion colony forming units (CFUs) of *Lactobacillus paracasei* CNCM I-1572 and 5 billion CFUs of *L. paracasei* LPCS01) produced greater total concentrations of leucine, branched-chained amino acids (BCAAs), and EAA following consumption of a pea protein isolate compared to a placebo [20]. Another study reported an improvement in maximum EAA and total amino acid (TAA) concentrations following 14 days of *Bacillus coagulans* GBI-30, 6086 combined with a milk protein isolate [21]. While the effects of probiotics are often shared by those of the same genus (e.g., *Bacillus*), many effects are strain-specific, and it is important to expand on these early findings to determine which probiotic strains may improve protein kinetics [22]. Additionally, though whey protein is generally considered the standard with regards to protein quality and is the most widely consumed protein by athletes [23,24], no data exists regarding the effects of probiotics on whey protein digestion. As such, elucidating the effects of probiotics on whey protein digestion would be of great interest to researchers, clinicians, and athletes alike.

Therefore, the purpose of this study was to evaluate the influence of 28 days of DE111 supplementation on plasma amino acid concentrations after consumption of a whey protein beverage in healthy adult men and women. A secondary aim of the study was to observe potential changes in body composition following the probiotic intervention. 

## 2. Materials and Methods 

Twenty-two recreationally active men (*n* = 11; 24.3 ± 3.2 yrs; 89.3 ± 7.2 kg; 1.83 ± 0.06 m; 17.4 ± 4.2% body fat) and women (*n* = 11; 23.0 ± 2.8 yrs; 70.2 ± 15.2 kg; 1.68 ± 0.10 m; 22.4 ± 8.45% body fat) participated in this double blind, placebo-controlled, randomized study. Following an explanation of all procedures, risks, and benefits, each participant provided their written informed consent prior to participation in this study. The research protocol was approved by the Institutional Review Board of Lipscomb University prior to participant enrollment. Exclusion criteria included the use of probiotic supplementation, ergogenic aids, or suffering from any medical, muscular, or metabolic contraindications.

### 2.1. Study Protocol

Participants reported to the Human Performance Lab (HPL) on two separate occasions at the beginning (PRE) and end (POST) of a 28-day supplementation intervention following a 10-h overnight fast. On each testing visit to the lab, participants completed baseline body composition assessment and then consumed a 25-g bolus of whey protein (True Nutrition, Vista, CA, USA) dissolved in 400 mL of water (Table 1). Following drink consumption, blood samples were obtained from an antecubital catheter before (0 min) and every 15 min following protein consumption for 120 min (7 draws total) and were later analyzed for plasma leucine, BCAA, EAA, and TAA. Following the PRE testing visit, participants were randomly assigned to a probiotic (PROB; *n* = 11) or placebo (PL; *n* = 11) group and completed 28 days of supplementation before returning to the lab for their POST testing visit. Additionally, participants were instructed to report to the lab hydrated while abstaining from caffeine, alcohol, and vigorous exercise for at least 24 h prior to both laboratory testing sessions. For the 2-h duration following consumption of the drink, participants abstained from drinking any water or other liquid.

### 2.2. Body Composition Analysis

Lean body mass, fat mass, and percent body fat were estimated using multi-frequency bioelectrical impedance analysis (BIA) using the InBody^®^ 570 Body Composition Analyzer device (Biospace, Inc., Seoul, Korea). Body composition from BIA is obtained from the measures of resistance and reactance when an electrical current travels through the body. Prior to each assessment, the participants’ hands and feet were thoroughly cleaned with InBody^®^ provided tissues. Age, height, and sex were manually entered, while a scale positioned within the device assessed body mass. The participant was then instructed from the software to stand fully erect on the measurement electrodes situated on the platform and to hold hand electrodes, with arms extended, without touching the sides of their body. Participants were asked to refrain from moving or talking until the assessment was completed. 

### 2.3. Blood Collection, Handling, and Storage 

Blood samples during the experimental trials (0–120 min) were drawn from a 20-g Teflon^TM^ cannula placed in a superficial forearm vein using a three-way stopcock with a male Luer lock adapter. The cannula was maintained patent using an isotonic saline solution, and blood was drawn from the cannula with a plastic syringe prior to drink consumption (0 min), and at 15-, 30-, 45-, 60-, 75-, 90-, 105-, and 120-min post-consumption (15 min, 30 min, 45 min, 60 min, 75 min, 90 min, 105 min, 120 min, respectively).

Blood samples were drawn into heparin-treated Vacutainer tubes and were centrifuged immediately for 15 min at 1500× *g* at 4 °C. The resulting plasma was aliquoted and stored at −80 °C until analysis. Samples were thawed only once for biochemical analysis. 

### 2.4. Supplementation Protocol

Both the PROB and PL groups completed daily supplementation for 28 days. The PROB supplement consisted of one billion CFUs of *Bacillus subtilis* DE111, (DE111^®^, Deerland Enzymes, Kennesaw, GA, USA), similar to previous investigations [12,13]. The placebo capsules consisted of maltodextrin, and participants consumed their respective treatment (PROB or PL) in the form of a capsule, with both treatments being identical in appearance. At the beginning of each week of the 4-week intervention, participants were provided their respective supplements in individual labeled bags and were required to consume their supplement with a normal meal. The subsequent week, participants would return used bags to track compliance before they were provided with their supplement for the following week.

### 2.5. Dietary Logs

Participants were instructed to maintain their normal dietary intake leading up to experiment trials. Participants completed a 3-day dietary food log for all food and beverages consumed leading up to lab visits before and after the 28-day intervention. Dietary food logs consisted of the day before the experimental trial and 2 additional days (1 weekday, 1 weekend day). Participants were instructed not to eat or drink anything within 10 h of reporting to the laboratory for experimental trials. Dietary food logs were used to provide an estimate of total kilocalorie intake (kcal) and macronutrient distributions (carbohydrate, protein, and fat) of the participant’s typical weekly diet. All dietary analysis was completed using the MyFitnessPal application (Under Armour Inc., Baltimore, MA, USA), which contains a large, detailed US-branded food database.

### 2.6. Plasma Amino Acid Analysis 

#### 2.6.1. Materials

Cell-free solid 20 amino acid (AA) mix of non-labeled standards, STD-AA (ULM-7891, Lot: PR-28676), and ^13^ C, ^15^N–isotopically labeled AA internal standard mix, IS-AA (CNLM-6696: U-13C, 97–99%+; U-15N, 97–99%, Lot: PR-22794), were purchased from Cambridge Isotope Labs, Inc. (Tewksbury, MA, USA) and stored at 4°C. LC/MS grade acetonitrile, water, ammonium acetate and formic acid were purchased from Fisher Scientific (Waltham, MA, USA).

#### 2.6.2. Sample Preparation

The extraction solution for all plasma samples consisted of 3:1 ACN:H_2_O with 10 mM ammonium acetate and 0.1 (v)% formic acid including the IS-AA mix. Plasma samples were extracted in a 1:9 ratio of plasma:extraction solution, vortexed for 10 s, and centrifuged at 15,000× *g* for 10 min [25]. Supernatant was transferred to a 96-well plate, then sealed with clear film covers to prevent evaporation.

A study pool quality control (SPQC) sample was created by mixing an equal volume aliquot from each unknown sample. All calibration standards (C1–C7) were prepared in a composite plasma sample taken from healthy volunteers for matrix matching (detailed calibrator preparation information in Supplementary Materials). A true blank was prepared by extracting calibrator plasma matrix using the same extraction solvent with no IS-AA mix. Calibrators C1–C4 were used as the quality control samples.

#### 2.6.3. LC/MS Data Acquisition and Processing

Hydrophilic interaction chromatography (HILIC) separation of calibrators and experimental samples was performed on a Waters Acquity H-Class UPLC instrument equipped with a quaternary pump and an Acquity UPLC^®^ BEH Amide column (2.1 × 100 mm, 1.7 µm particle size, part 186004801) including a Van Guard™ UPLC BEH Amide pre-column (2.1 × 5 mm, 1.7 µm particle size, part 186004799). Column temperature was 35.0 °C, the autosampler was kept at 10.0 °C, injections were 1 µL, and the solvent flow rate was 0.4 mL/min. The UPLC was inline with a Waters Xevo TQD (triple quadrupole) ZSpray ESI (electrospray ionization) mass spectrometer, which was controlled by MassLynx software (version 4.2). The mass spectrometer was operated in ESI-positive mode, at a capillary voltage of 1.50 kV, with a desolvation temperature of 350 °C, a desolvation gas flow of 650 L/hr, a cone source gas flow of 0 L/hr, and a source temperature of 150 °C. Gradient separation was performed using the following solvents: channel A: water, B: acetonitrile, C: 500 mM ammonium acetate in water, and D: 5 (V)% formic acid in water [25]. The column was equilibrated for at least 4 min in the starting conditions before use and after every eight samples an acetonitrile solvent blank was run.

Multiple reaction monitoring (MRM) was used to quantify the amino acid content in the samples. The IS-AAs had identical retention times as the non-isotopically labeled STD-AAs. Dwell times were automatically selected at 0.005 s. Results from histidine and cysteine quantification were not reproducible; they are omitted from these tables. 

Following acquisition, data files were imported into Skyline Targeted Mass Spec Environment for processing [26]. Retention time matching was manually inspected, and a seven-point bilinear regression weighted 1/x^2^ calibration was created for each AA (0.2 to 26.2 µg/mL global range; see Supplementary Materials for calibration range of individual AAs) [27] The AA concentration of each sample was calculated relative to the peak area for isotopically labeled internal standard, similar to previous work [28].

#### 2.6.4. Quality Control of LC/MS Data

When data processing in Skyline was completed, data were further analyzed to determine if the plate met pre-set quality control (QC) criteria. The following criteria were required to meet QC: *R*^2^ value above 0.95 with a minimum of 6 points in the calibration curve, 75% of the non-zero calibrators within ±15% of the theoretical concentration, except the lower limit of quantitation (LLOQ) should be within ±20% [29]. Additional details on the calibration range, limit of quantitation, and QC results are provided in the Supplementary Materials.

### 2.7. Statistical Analysis

Prior to hypothesis testing, the Shapiro–Wilk test was used to evaluate the assumption of normality for dependent variables at PRE 0 min. In the event that the PRE 0 min data was not normally distributed, we opted to log-transform the measurements (using the natural logarithm). Body composition variables were analyzed via a three-way (supplementation (PRE, POST), group (PROB, PL), and sex (male, female) repeated measures analysis of variance (ANOVA). To examine group and intervention differences in the leucine, BCAA, EAA, and TAA responses to whey consumption before and following the 28-day supplement intervention, a three-way (supplementation (PRE, POST) × time (0 min–120 min) × group (PROB, PL)) repeated measures ANOVA was performed. Additionally, at PRE and POST, a time × group × sex repeated measures ANOVA was conducted to determine if there was any effect of sex on the amino acid response. The effect of the intervention on AUC measures, calculated using the trapezoidal methods, was examined using a three-way (supplementation (PRE, POST) × group (PROB, PL) × sex (male, female)) repeated measures ANOVA. Significant interactions were decomposed with follow-up repeated measures ANOVAs, Bonferroni-corrected dependent samples *t* test, and/or independent samples *t*-test on the simple main effects. Significant main effects that were not involved in an interaction were analyzed with Bonferroni-corrected dependent samples *t*-test on the marginal means. For all analyses, a criterion alpha level of *p* ≤ 0.05 was considered significant. Statistical analyses were performed using IBM SPSS version 22.0 (Armonk, NY, USA).

## 3. Results

### 3.1. Participant Characteristics

Twenty-four participants were initially recruited for this investigation, of which two were removed before analysis (PROB = 1, PL = 1), for a final of 22 participants in the analysis (Table 1). The two participants were not able to complete post testing due to unresolvable scheduling conflicts and discontinued supplementation during the 28-day supplementation period due to the time commitment. There were no significant differences (*p* > 0.05) in baseline characteristics of the remaining 22 participants. No significant differences (*p* = 0.67) were observed between groups for supplement compliance between the PL (98.7 ± 1.8%) and PROB (99.0 ± 1.7%). Additionally, no significant differences (*p* > 0.05) in dietary composition (protein, carbohydrates, fats, total kcals) were noted between the groups throughout the 28-day protocol. Both supplements were well tolerated, and no adverse events were reported by participants.

There was no main effect for time observed between PROB and PL for body mass (*p* = 0.116), body fat percentage (*p* = 0.648), lean body mass (*p* = 0.361), fat mass (*p* = 0.287), or TBW (*p* = 0.278). Additionally, no group by time interactions (*p* > 0.05) were found for body mass, body fat percentage, lean body mass, fat mass, or TBW (Table 2). There was no supplementation × group × sex interaction for any body composition variable (all *p* > 0.05)

### 3.2. Plasma Amino Acid Analysis

**Figure 1:**
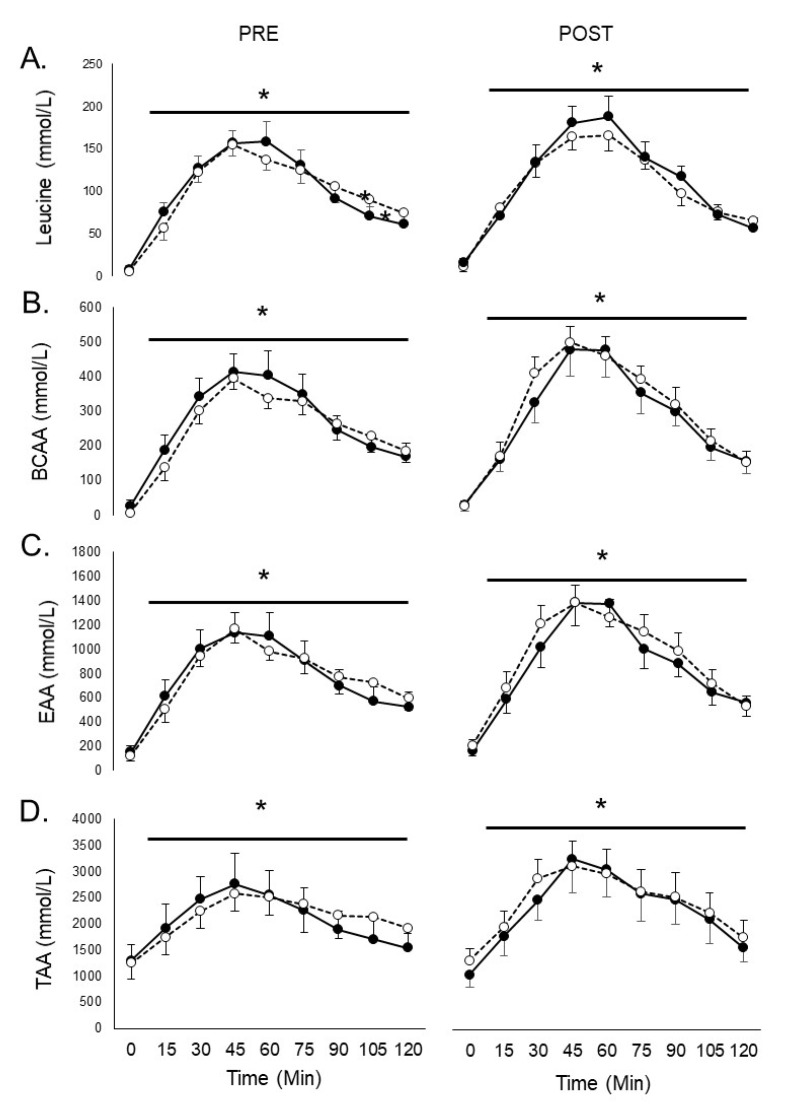
Plasma leucine (**A**), branched-chain amino acids (BCAAs; **B**), essential amino acids (EAAs; **C**), and total amino acids (TAAs; **D**) from 0–120 min following consumption of 25 g of whey protein before (PRE) and after (POST) a 28-day supplement intervention. Groups: PROB: *Bacillus subtilis* DE111; PL: placebo. * and horizontal bar indicate a main effect of time for both groups. Values represent means ± *SEM*s.

Following consumption of the whey protein beverage, plasma leucine, BCAA, EAA, and TAA concentrations were significantly elevated (*p* < 0.05) at all subsequent time points (15–120 min) PRE and POST intervention (Figure 1). However, the repeated measures ANOVA revealed no group × supplementation × time interaction for leucine (*p* = 0.504), BCAA (*p* = 0.602), EAA (*p* = 0.494), or TAA (*p* = 0.638). There were no significant group × supplementation interactions found for leucine (*p* = 0.675), BCAA (*p* = 0.414), EAA (*p* = 0.635), or TAA (*p* = 0.824). No group × time interactions were observed for leucine (*p* = 0.687), BCAA (*p* = 0.810), EAA (*p* = 0.639), or TAA (*p* = 0.790). Finally, no time × supplementation interactions were seen for leucine (*p* = 0.069), BCAA (*p* = 0.085), EAA (*p* = 0.088), or TAA (*p* = 0.136). At PRE and POST, there was no significant group by time by sex interactions for the amino acid response for leucine, BCAA, EAA, and TAA (all *p* > 0.05).

**Figure 2:**
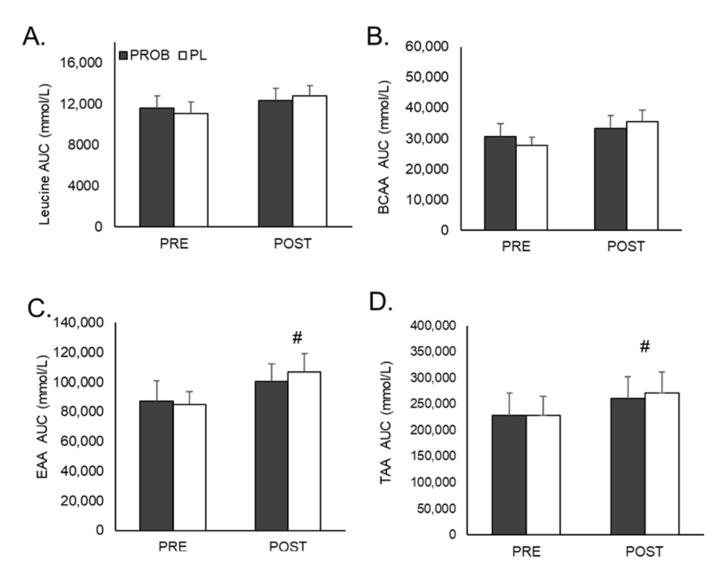
Area under the curve (AUC) values for plasma leucine (**A**), branched-chain amino acids (BCAAs; **B**), essential amino acids (EAAs; **C**), and total amino acids (TAAs; **D**) from 0–120 min following consumption of 25 g of whey protein before (PRE) and after (POST) a 28-day supplement intervention. Groups: PROB: *Bacillus subtilis* DE111; PL: placebo. # main effect observed with higher values at POST. Values represent means ± *SEM*s.

No time by group interaction was observed for AUC for leucine (*p* = 0.524), BCAA (*p* = 0.345), EAA (*p* = 0.512), or TAA (*p* = 0.712) (Figure 2). There was a main effect for time for EAA (*p* = 0.013) and TAA (*p* = 0.024), with greater AUC values observed at POST for EAA (mean difference: 17,832 ± 6521.7; 95% confidence interval: 4228.4, 31,436.3) and TAA (mean difference: 37,632.9 ± 15,430.8 mmol/L; 95% confidence interval: 5444.8, 69,820.9) with groups collapsed. No other main effects for time were observed. Additionally, there was no significant group × supplementation × sex interactions for AUC concentrations for leucine, BCAA, EAA, and TAA (all *p* > 0.05).

## 4. Discussion

The main purpose of this study was to evaluate the effects of 28 days of probiotic supplementation on the plasma amino acid response to whey protein ingestion in healthy recreationally active adults. Our findings showed no significant difference in the rate or magnitude of leucine, BCAA, EAA, and TAA in the 2 h following whey protein ingestion between those that supplemented with PROB or PL. Furthermore, 28 days of PROB administration resulted in no significant differences in body composition between groups. 

In the present study, we were unable to demonstrate that chronic *Bacillus subtilis* DE111 probiotic supplementation resulted in improved amino acid response to whey protein feeding. Jager et al. [20] recruited 15 active men in a cross-over study design that required participants to consume 20 g of pea protein or pea protein with an added probiotic (5 billion CFU of *L. paracasei* CNCM I-1572 and 5 billion CFU of *L. paracasei* LPCS01) for two weeks leading up to an acute feeding of each condition with subsequent plasma amino acid measurements. Resulting data revealed increased maximum concentrations and AUC values for leucine, BCAA, and total EAA in the pea protein + probiotic treatment, while the time to reach maximum concentration was no different between treatments. In agreement with our findings, *Bacillus coagulans* GBI-30, 6086, consumed in conjunction with a milk protein isolate, did not alter AUC values or time to maximum concentration for leucine, BCAA, EAA, or TAA in healthy males and females compared to placebo [21]. However, peak concentrations for leucine, BCAA, and TAA were higher following the probiotic treatment in that study. While the present study was designed to determine if a probiotic may improve whey protein digestion and amino acid absorption, the discrepancies with previous work are likely explained by the type of protein ingested and strain-specific effects of probiotics. Whey protein is among the quickest and most easily digested proteins, demonstrating a superior uptake into the plasma compared to other protein types (e.g., soy, casein) [23]. However, tactics to improve the rate or magnitude of whey protein absorption via hydrolysis and other modifications have proven to be equivocal [30,31,32]. Additionally, while many of the beneficial effects of probiotics are shared by those of the same genus [22,33], strain-specific benefits exist, and much more data is needed to determine which probiotics are optimal for improvements in digestion. Based on the current study and previous work, it is likely that those who consume primarily lower-quality, slow-release, or plant-based proteins may benefit the most from the potential effects of probiotics on aminoacidemia. As such, future chronic training studies recruiting vegetarian or vegan athletes may shed light on the practical application of certain probiotics in these populations. Concerning DE111, future work should examine the effects of DE111 supplementation on the amino acid response to a variety of protein types (e.g., whey, soy, pea, egg) while utilizing more sophisticated methodology, including measurement of muscle protein synthesis (MPS). Finally, a primary mechanism of probiotics, and specifically DE111, is their ability to maintain intestinal integrity and reduce inflammation, which may only be challenged during an acute exercise stressor. Previous work indicates that acute resistance exercise results in splanchnic hypoperfusion to the gut, resulting in gut damage and reduced protein absorption [34]. Thus, studies implementing peri-workout protein consumption while measuring amino acid uptake and MPS would increase our knowledge of the efficacy of probiotics for the athletic population.

While it was not the primary focus of this study, we observed no differences in body composition between groups following the supplementation period. This is not surprising, given the short intervention period. In previous work from our lab, we observed improved body fat percentage following 10 weeks of offseason training in female division I athletes who supplemented with 5 billion CFU/day of DE111 [14]. However, these findings were not replicated in a study featuring a sample of leaner college baseball players, which found no effect of daily 1 billion CFU of DE111 supplementation on measures of body composition following 12 weeks of offseason training [13]. Thus, it is possible that a higher dose of DE111 is needed to observe improvement in body fat percentage in conjunction with exercise. Utilizing a study design most similar to the present study, Antonio and colleges [35] enrolled 6 males and 14 females in a 6-week study where they were provided either a probiotic (5 billion CFU Bifidobacterium BR03 and 5 billion *Streptococcus thermophilus* FP4) or placebo. Participants were instructed to maintain their normal exercise and diet routine, and following 6 weeks of supplementation, there were no differences in body weight, lean body mass, fat mass, bone mineral content, body fat percentage, or abdominal fat mass [35]. It is important to note that the effects of probiotics are strain-specific, and therefore we cannot conclude that probiotic supplementation has no effect on body composition in active adults based on the existing literature. Studies in obese individuals show positive effects of improved fat loss in as little as 4 weeks [36]. For active, lean individuals, a longer intervention with participants adopting a caloric deficit would provide a better indication as to the effect of probiotics on body composition.

## 5. Conclusions

In summary, 28 days of DE111 supplementation did not result in improved plasma amino acid levels of leucine, BCAA, EAA, or TAA in healthy recreationally active adults. As the effects of probiotics are strain-specific, there is much more work to be done in this area to elucidate the effects of probiotic supplementation on protein utilization in exercising individuals.

## Figures and Tables

**Table 1:** Amino acid composition of whey protein supplement (Supplementary Materials).

Amino Acid	g/100 g
Alanine	3.5
Arginine	2.3
Aspartic Acid	8.4
Cystine	1.7
Glutamic Acid	13.3
Glycine	1.4
Histidine	1.6
Isoleucine	4.6
Leucine	8.8
Lysine	7.5
Methionine	1.6
Phenylalanine	2.6
Proline	6.6
Serine	4.6
Threonine	4.5
Tryptophan	1.3
Tyrosine	2.3
Valine	4.4

**Table 2:** Body Composition Values Before and After Supplementation Period.

Variable	Group	Pre	Post	Group × Time
Body Mass (kg)	PROB	83.1 ± 17.7	83.7 ± 18.1	*p* = 0.346
	PL	74.6 ± 12.2	74.8 ± 12.0	
Lean Body Mass (kg)	PROB	65.5 ± 17.0	65.6 ± 17.0	*p* = 0.785
	PL	60.7 ± 11.2	60.9 ± 11.0	
Fat Mass (kg)	PROB	17.6 ± 6.9	18.1 ± 7.5	*p* = 0.168
	PL	13.9 ± 4.7	13.9 ± 4.9	
Body Fat (%)	PROB	21.6 ± 8.3	21.9 ± 8.9	*p* = 0.243
	PL	18.8 ± 5.9	18.6 ± 6.1	
Total Body Water (kg)	PROB	47.7 ± 12.4	47.8 ± 12.5	*p* = 0.883
	PL	44.3 ± 8.2	44.5 ± 8.0	

Data presented as mean ± *SD*. PROB: probiotic; PL: placebo.

## Data Availability

Data from this study is available upon reasonable request.

## Supplementary Materials

The following are available online at https://www.mdpi.com/2411-5142/6/1/1/s1: Amino acid analysis supplement.
