# Supplementary material for: Plasma Amino Acid Response to Whey Protein Ingestion Following 28 Days of Probiotic (Bacillus subtilis DE111) Supplementation in Active Men and Women
Source: J Funct Morphol Kinesiol. 2020 Dec 23;6(1):1. doi: 10.3390/jfmk6010001 (PMC7838959; doi:10.3390/jfmk6010001)
Supplement: Supplementary file 1 [file jfmk-06-00001-s001.pdf]

Experimental Methods

Stock Standards Preparation: The stock STD-AA (48.4 mg) and IS-AA (66.7 mg) standard solutions were dissolved in 10.00 mL of 50/50 ACN/H_2O with 0.1 (v)% formic acid, vortexed, and filtered with a 0.2 µm filter. These standard solutions were clear, slightly brown in color, and stored in a -20 ℃ freezer. Table S1 lists the amino acid mass percentages by weight from each mixture and the stock concentrations. Results from histidine and cysteine quantification were not reproducible, they are omitted from the table.

Table S1: Amino acid content as weight mass percent and stock concentrations of STD-AA or IS-AA mixtures.

Amino Acid	STD-AA mass %	IS-AA mass %	Stock STD-AA ug/mL	Stock IS-AA ug/mL
**Ala - alanine**	5.9	7.5	245	404
**Arg - arginine**	2.8	6.6	116	355
**Asn - asparagine**	5.1	4.7	212	253
**Asp – aspartic acid**	8.2	7.9	341	425
**Gln - glutamine**	5.1	5.4	212	291
**Glu – glutamic acid**	0.7	6.9	29.1	371
**Gly - glycine**	4.5	3.7	187	199
**Ile - isoleucine**	1.3	6	54.0	323
**Leu - leucine**	5.8	10.4	241	560
**Lys - lysine**	9.8	8.2	407	441
**Met - methionine**	3.6	1.5	149	80.7
**Phe - phenylalanine**	5.5	7.3	228	393
**Pro - proline**	4.1	1.9	170	102
**Ser - serine**	2.9	2.3	120	124
**Thr - threonine**	11.8	4.1	490	221
**Trp - tryptophan**	3.3	3	137	161
**Tyr - tyrosine**	4.9	4.5	203	242
**Val - valine**	7.4	6.6	307	355

Calibrator Preparation: Calibrator solutions (C1 -C7) of STD-AA were prepared with serial dilution (concentration for individual amino acids in Table S2). C1 was the stock STD-AA solution listed in Table S1 and C2-C4 are serial dilutions starting with C1 of 500 µL in 1000 µL. C5 was 100 µL of C1 in 1000 µL and C6-C7 are serial dilutions starting from C5 of 500 µL in 1000 µL. To create the calibrator standards in the plasma matrix, 40 µL of the C1-C7 calibrator solutions was added to 600 µL of IS-ES, and 80 µL of plasma matrix and their final concentrations are listed in Table S2. Post preparation of the calibrator stock solutions, the extraction solution was appropriately diluted to account for the solvent introduced in the calibrator samples such that the experimental samples would have the same final concentration of internal standards.

Table S2: Final concentrations of standard amino acid (STD-AA) and isotopically labeled amino acid internal standards (IS-AA) when diluted with plasma. STD-AA concentration is for the calibration series (C1-C7) and IS-AA is for both the calibration series and the experimental samples.

Amino Acid	STD-AA C1 ug/mL	STD-AA C2 ug/mL	STD-AA C3 ug/mL	STD-AA C4 ug/mL	STD-AA C5 ug/mL	STD-AA C6 ug/mL	STD-AA C7 ug/mL	IS-AA ug/mL
**Ala**	13.6	6.81	3.40	1.70	1.36	0.681	0.340	2.36
**Arg**	6.46	3.23	1.61	0.808	0.646	0.323	0.162	2.07
**Asn**	11.8	5.88	2.94	1.47	1.18	0.588	0.294	1.48
**Asp**	18.9	9.46	4.73	2.36	1.89	0.946	0.473	2.48
**Gln**	11.8	5.88	2.94	1.47	1.18	0.588	0.294	1.70
**Glu**	1.61	0.81	0.404	0.202	0.162	0.081	0.040	2.17
**Gly**	10.4	5.19	2.60	1.30	1.04	0.519	0.260	1.16
**Ile**	3.00	1.50	0.750	0.375	0.300	0.150	0.075	1.88
**Leu**	13.4	6.69	3.35	1.67	1.34	0.669	0.335	3.27
**Lys**	22.6	11.3	5.65	2.83	2.26	1.13	0.565	2.58
**Met**	8.31	4.15	2.08	1.04	0.831	0.415	0.208	0.471
**Phe**	12.7	6.34	3.17	1.59	1.27	0.634	0.317	2.29
**Pro**	9.46	4.73	2.36	1.18	0.946	0.473	0.237	0.597
**Ser**	6.69	3.35	1.67	0.836	0.669	0.335	0.167	0.722
**Thr**	27.2	13.6	6.81	3.40	2.72	1.36	0.681	1.29
**Trp**	7.61	3.81	1.90	0.952	0.761	0.381	0.190	0.942
**Tyr**	11.3	5.65	2.83	1.41	1.13	0.565	0.283	1.41
**Val**	17.1	8.54	4.27	2.13	1.71	0.854	0.427	2.07

Detailed Data Processing: All the data for each plate was imported into Skyline at the same time, with individual plates each correlated with an individual data file. Once all compounds were checked and retention times were adjusted as needed, the plate was rescored to update peak areas for further analysis. This process was repeated until retention times no longer needed adjustment.

The calibration curve for this assay used a bilinear regression which allows for more data points at the cost of greater processing power, and the $1/x^2$ weighting is most appropriate for bioanalytical LC MS/MS [1]. The bilinear regression calculates a linear response above a turning point, a flat response below the turning point, and the turning point (TP) may be considered the limit of detection (LOD) for bilinear calibration curves. Points were removed from the calibration curve starting with the least accurate samples until the R^2 value per compound was 0.95 or greater, and the points remaining were within ±20% of the theoretical concentration. The

LOD was calculated as concentration of the blank plus 3 times the standard deviation of the blank external standards. The limit of quantitation (LOQ) was determined to be the lowest concentration value that fit within the parameters of the maximum bias and maximum coefficient of variation (CV). The max LOQ bias is the maximum allowed difference from the calibration curve and was set at 15%, while the max LOQ CV is the maximum allowed CV of the peak areas of the set of internal standards (IS) with a specified concentration and was set to 15%.

The LLOQ was determined to be the highest value between the Skyline determined LOD, LOQ, and TP, and is usually the LOQ. For the QC samples with known concentrations, they were divided into 4 levels, Lower limit QC (LLQC), Lower QC (LQC), Medium QC (MQC), and High QC (HQC). At least 50% of the QC's per level and 67% of the overall QCs needed to be within 15% of the theoretical concentrations. 50% of the sample pool QCs needed to be within ±50% of the average concentration of the sample pool QC. Each analyte per plate was analyzed separately to determine if it can be accurately quantitated on the plate, then these analyte values were averaged to determine plate data quality.

Table S3: Average values per compound across all plates for R^2, Working range, QC accuracy, and other figures of merit.

Amino Acid	*R^2*	*Working Max (µg/mL)*	*Working Min (µg/mL)*	*% of Known QC within 15%*	*# of points used*	*LOD (µg/mL)*	*LOQ (µg/mL)*	*TP (µg/mL)*
arginine	0.988	6.5	0.5	82%	11	<0.01	0.49	0.0002
lysine	0.990	22.6	1.3	89%	12	<0.01	1.35	0.19
glycine	0.981	10.4	2.1	49%	8	<0.01	2.13	0.22
alanine	0.984	13.6	0.7	76%	12	<0.01	0.73	0.26
serine	0.989	6.7	0.9	55%	8	<0.01	0.92	-0.07
proline	0.985	9.5	1.1	73%	10	<0.01	1.12	0.29
valine	0.991	16.5	1.4	73%	10	<0.01	1.45	0.43
threonine	0.988	26.2	1.9	76%	12	<0.01	1.86	0.60
isoleucine	0.990	5.4	0.4	82%	11	<0.01	0.40	0.08
leucine	0.991	12.3	0.5	94%	13	<0.01	0.50	0.01
asparagine	0.988	11.9	1.3	62%	10	<0.01	1.29	0.22
glutamine	0.981	11.8	4.9	19%	6	0.44	4.93	0.51
glutamic acid	0.991	1.6	0.2	71%	11	<0.01	0.20	0.03
aspartic acid	0.985	18.9	1.8	79%	10	0.26	1.84	0.48
methionine	0.995	8.3	0.7	79%	11	<0.01	0.65	0.01
phenylalanine	0.984	12.7	4.9	37%	8	<0.01	4.89	0.27
tyrosine	0.980	11.3	2.7	35%	8	0.06	2.67	0.01
tryptophan	0.988	7.6	1.3	57%	9	<0.01	1.31	0.18

1. Gu, H.; Liu, G.; Wang, J.; Aubry, A.-F.o.; Arnold, M.E. Selecting the correct weighting factors for linear and quadratic calibration curves with least-squares regression algorithm in bioanalytical LC-MS/MS assays and impacts of using incorrect weighting factors on curve stability, data quality, and assay performance. *Anal. Chem.* **2014**, *86*, 8959-8966.
